# Correction: WDR82-binding long noncoding RNA *lncEry* controls mouse erythroid differentiation and maturation

**DOI:** 10.1084/jem.2021168803262025C

**Published:** 2025-04-04

**Authors:** Shangda Yang, Guohuan Sun, Peng Wu, Cong Chen, Yijin Kuang, Ling Liu, Zhaofeng Zheng, Yicheng He, Quan Gu, Ting Lu, Caiying Zhu, Fengjiao Wang, Fanglin Gou, Zining Yang, Xiangnan Zhao, Shiru Yuan, Liu Yang, Shihong Lu, Yapu Li, Xue Lv, Fang Dong, Yanni Ma, Jia Yu, Lai Guan Ng, Lihong Shi, Jing Liu, Lei Shi, Tao Cheng, Hui Cheng

Vol. 219, No. 4 | https://doi.org/10.1084/jem.20211688| March 22, 2022

The authors regret that, during the process of data collation prior to publication, the β-actin blotting of the Hbb-b1 sample in Fig. 9 E (left panel) was erroneously replicated from the β-actin blotting of the Hba-a2 sample in Fig. 8 H. The figure’s source data file is correct, and this error does not alter the findings in this article. The original and corrected figures are shown here.

The error appears in print and in PDFs downloaded before March 28, 2025.

**Figure:**
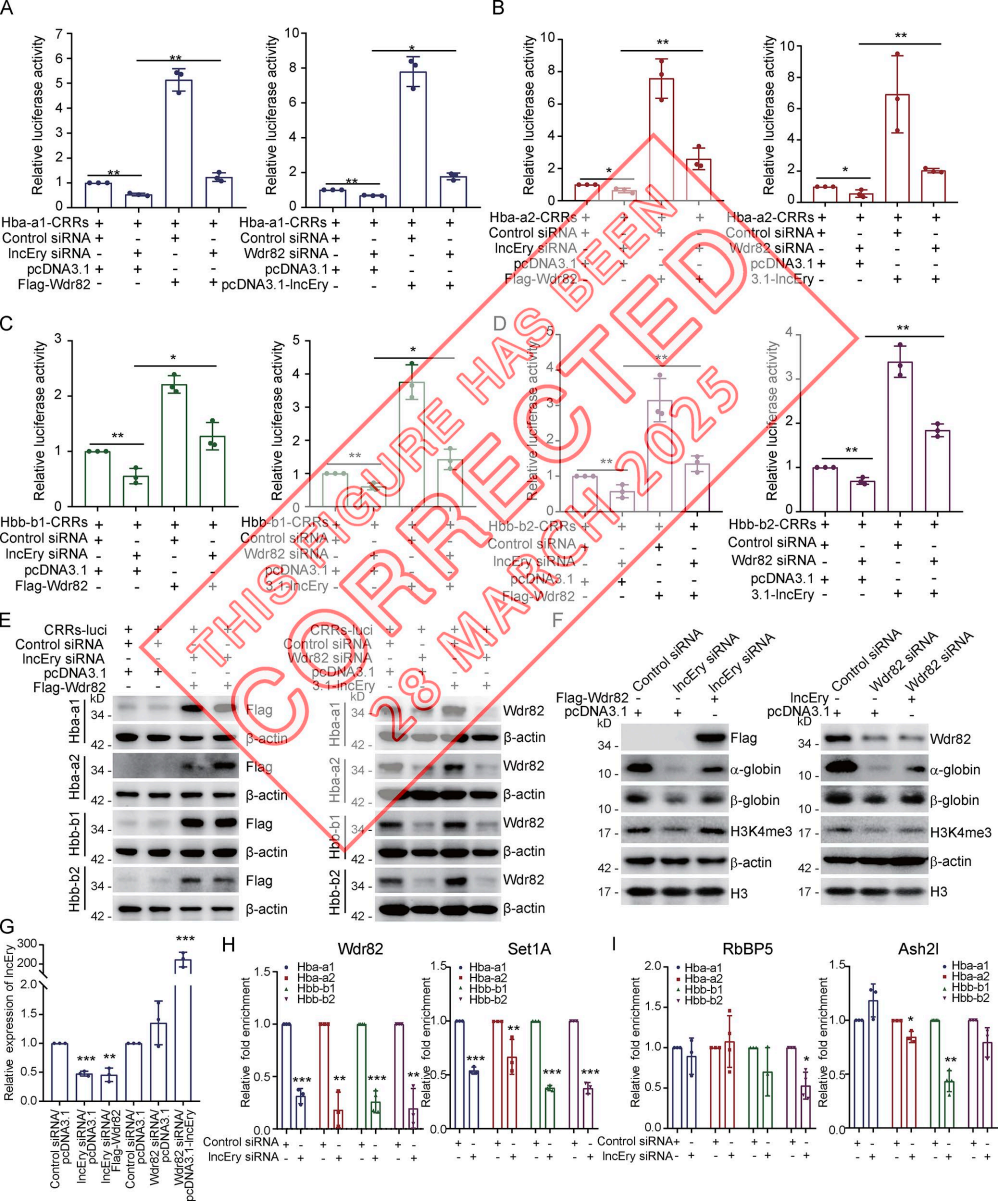


**Figure 9.**
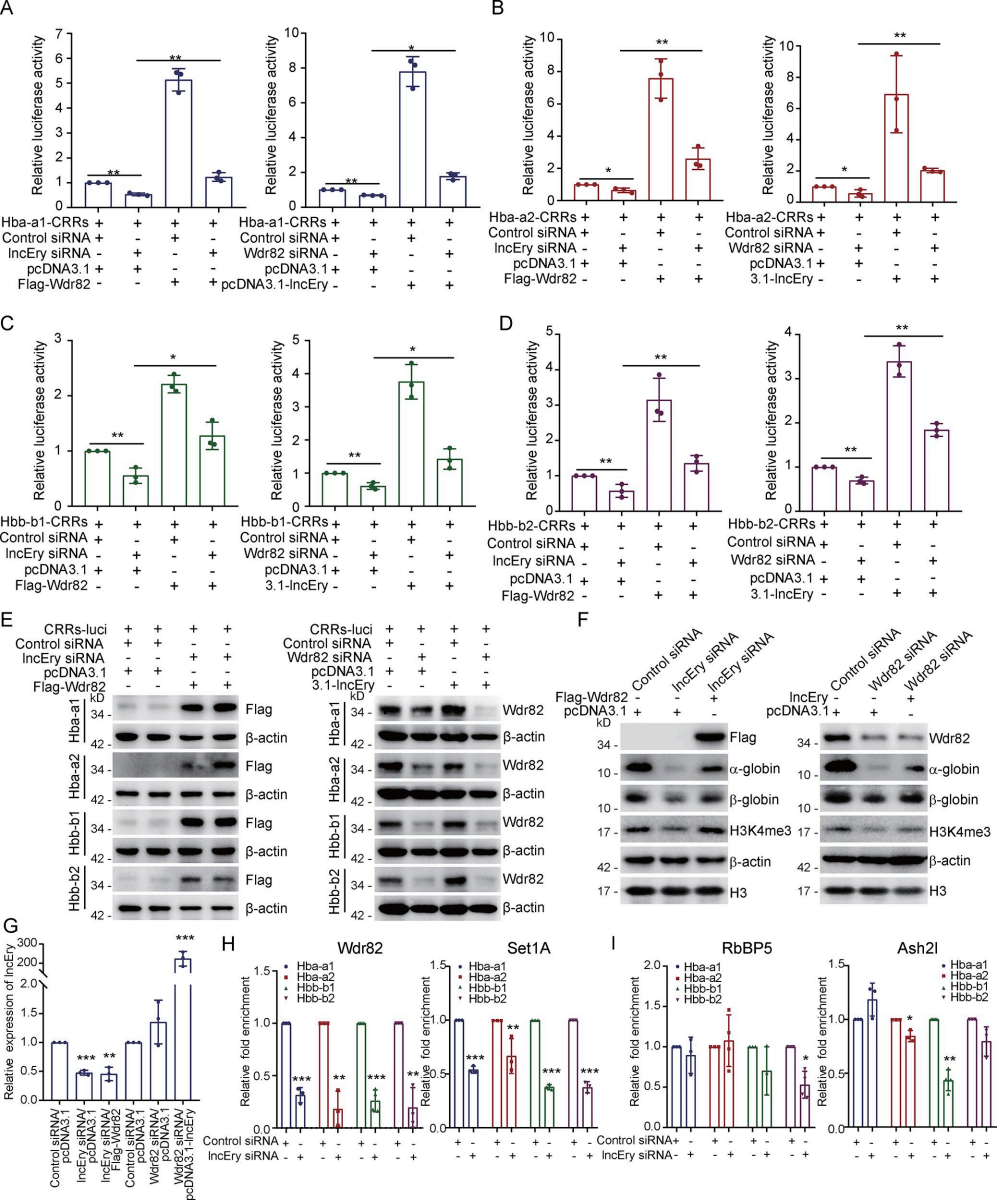
***lncEry*-Wdr82 regulates transcriptional activation of globin genes through CRRs. (A–D)** MEL cells were cotransfected with the indicated siRNAs or plasmids together with Renilla and the globin gene CRRs luciferase. The relative luciferase activity was determined by sequential normalization to Renilla and pGL3-vector activity (*n* = 3 samples). **(E)** Western blots of the expression of indicated proteins in the reporter assays shown in A–D. **(F)** MEL cells were cotransfected with the indicated siRNAs or plasmids, and the cellular extracts were analyzed by Western blotting with antibodies against the indicated proteins. **(G)** qRT-PCR of *lncEry* expression in each sample shown in F (*n* = 3 samples). **(H and I)** MEL cells were transfected with control or *lncEry* siRNAs; the soluble chromatin was immunoprecipitated with antibodies against Wdr82, Set1A (H), or RbBP5, Ash2l (I), and analyzed by qPCR with the indicated primers. The relative fold enrichment was determined by sequential normalization with the cycle threshold values of input and the control siRNA samples (*n* = 3 samples). Three to four independent experiments for A–I. Data are represented as mean ± SD. *, P < 0.05; **, P < 0.01; ***, P < 0.001; one-way ANOVA for A–D; unpaired two-tailed Student’s *t* test for G–I. Source data are available for this figure: SourceDataF9.

